# A Rare Tumor Causing Optic and Oculomotor Nerve Compression: Clivus Plasmacytoma Case Report

**DOI:** 10.7759/cureus.62447

**Published:** 2024-06-15

**Authors:** Betul Sevindik, Emine Uysal, Serdar Ugras

**Affiliations:** 1 Anatomy, Selcuk University Faculty of Medicine, Konya, TUR; 2 Radiology, Selcuk University Faculty of Medicine, Konya, TUR; 3 Pathology, Selcuk University Faculty of Medicine, Konya, TUR

**Keywords:** skull-base tumors, clivus tumors, cavernous sinus, clivus, intracranial plasmacytoma

## Abstract

Plasmacytomas rarely affect the skull base and may be found as an isolated lesion or as a part of multiple myeloma. The typical feature of plasmacytomas is aggressive bone destruction in the skull. It is often confused with the chordoma of the clivus. The most common location for skull-base plasmacytomas is the nasopharynx. The most commonly affected cranial nerve in clivus tumors is the abducens nerve. In our 64-year-old male case, a plasmacytoma was detected in the clivus. There was ptosis and decreased vision due to optic nerve and oculomotor nerve involvement due to the plasmacytoma. Radiotherapy was applied for the treatment.

## Introduction

Solitary plasmacytomas are rare and represent <1% of all head and neck tumors. A plasmacytoma refers to the accumulation of atypical plasma cells in a region outside the bone marrow. It can also be seen in solitary form as a subtype of plasma cell dyscrasias. It is a malignant tumor that can be a focal disease or the first manifestation of systemic multiple myeloma. It is called an intramedullary or extramedullary plasmacytoma depending on its relationship with the bone marrow. There are rare case reports in the literature regarding intracranial solitary plasmacytomas [1-3]. Extramedullary plasmacytomas are encountered in 5-10% of all plasma cell neoplasms [4]. It is necessary to make a differential diagnosis of solitary plasmacytomas, especially meningioma, chordoma, extraaxial tumor metastases, dural sarcomas, and lymphomas.

The differential diagnosis of plasmacytomas is based on histopathological studies. It is characterized by localized accumulation of neoplastic monoclonal plasma cells. The prognosis of solitary intramedullary plasmacytomas is considerably better than diffuse multiple myelomas. The typical feature of plasmacytomas is aggressive bone destruction in the skull on radiological examination [5]. Tumors and metastatic lesions can cause extensive damage to the clivus. The best known of these are clivus meningioma and clivus chordoma. In particular, these two groups are frequently confused with plasmacytomas, and it is necessary to distinguish them histopathologically [6]. Plasmacytomas involving the skull base are rare, and when the skull base is affected, they cause a variety of signs and symptoms that are usually nonspecific. Plasmacytomas can affect the function of some cranial nerves depending on the area of ​​the skull they affect. The affected cranial nerves are usually II, III, V, VI, and VII. Lateral medullary syndrome may develop as a result of the involvement of the posterior inferior cerebellar artery [3].

## Case presentation

**Figure 1:**
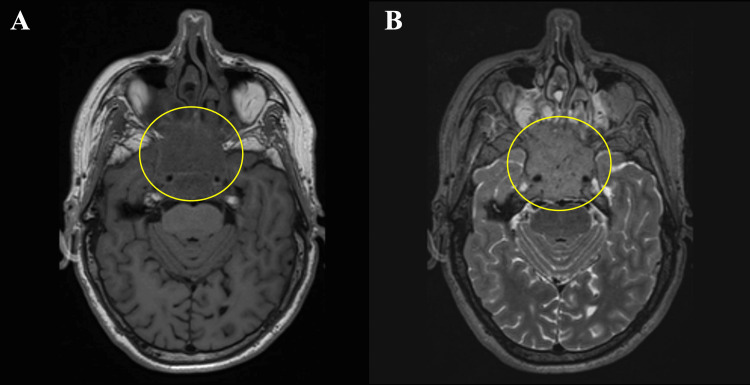
A: Tumor image in yellow circles on T1-weighted magnetic resonance imaging, B: tumor image in yellow circles on T2-weighted magnetic resonance imaging.

**Figure 2:**
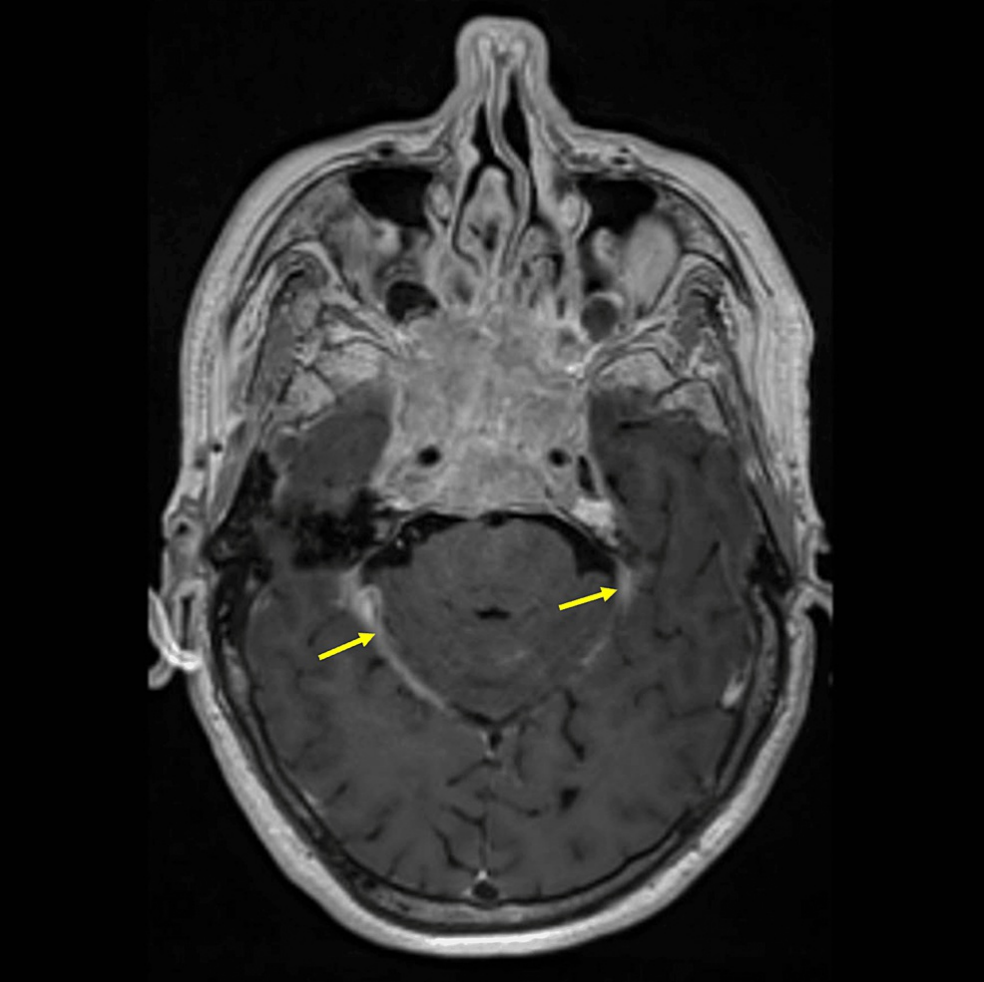
Thickening of the cerebellar tentorium on contrast-enhanced magnetic resonance imaging (indicated by yellow arrows).

**Figure 3:**
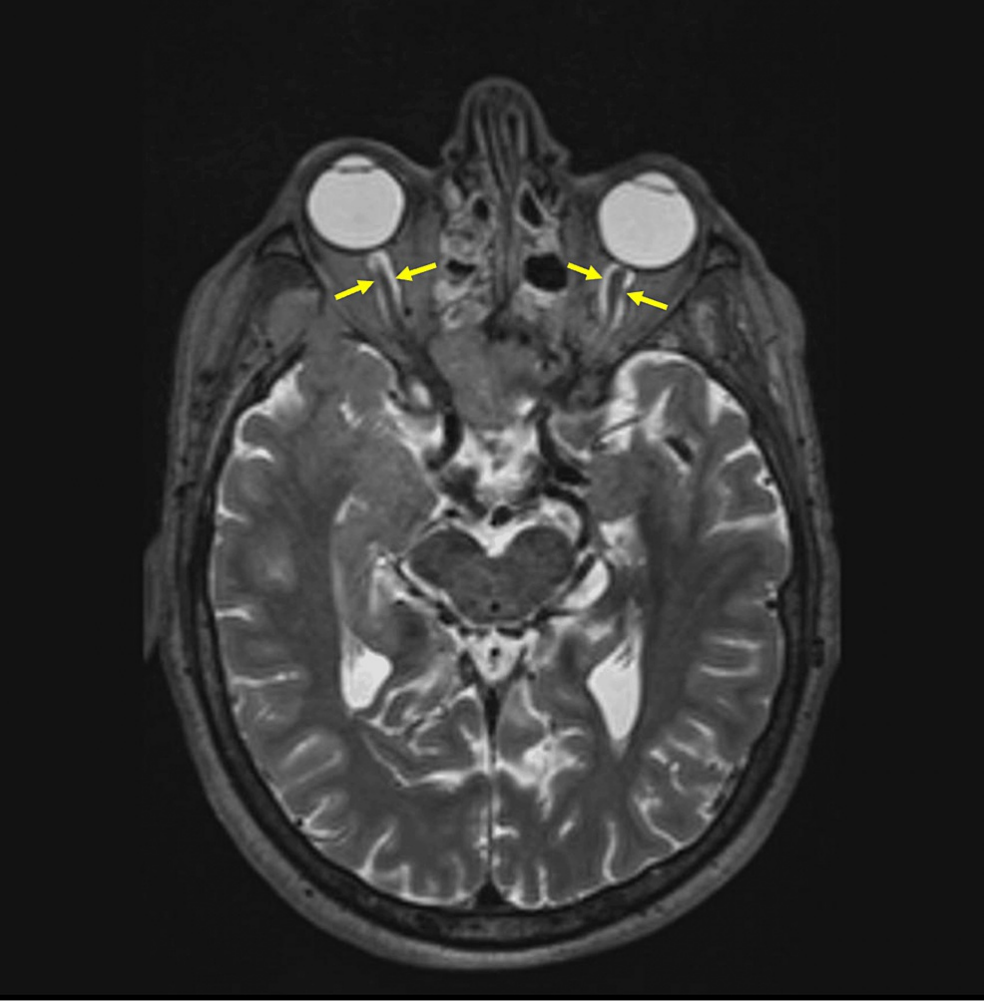
Increased fluid in the optic nerve sheath on T2-weighted magnetic resonance imaging (indicated by yellow arrows).

A 64-year-old male patient presented to the clinic with a headache, decreased visual function, and ptosis. The patient has no neurological deficit. There was no history of fever or vomiting. He has diabetes mellitus in his medical history. In the magnetic resonance imaging (MRI), a mass lesion measuring 44 x 44 x 32 mm, expanding the clivus, invading the bilateral cavernous sinus and pituitary gland, hyperintense relative to the brain parenchyma on the T2-weighted image, isointense relative to the brain parenchyma on T1 (Figure 1), and with intense contrast enhancement after intravenous contrast material injection, was observed. Ptosis occurs as a result of the lesion affecting the oculomotor nerve, located in the upper outer neighborhood of the cavernous sinus. There was thickening and contrast enhancement in the cerebellar tentorium on both sides (Figure 2). Increased perioptic fluid was detected in the optic nerve sheath (Figure 3). Increased fluid in the optic sheath appears as a result of the mass resulting from increased intracranial pressure. This situation also affects vision.

**Figure 4:**
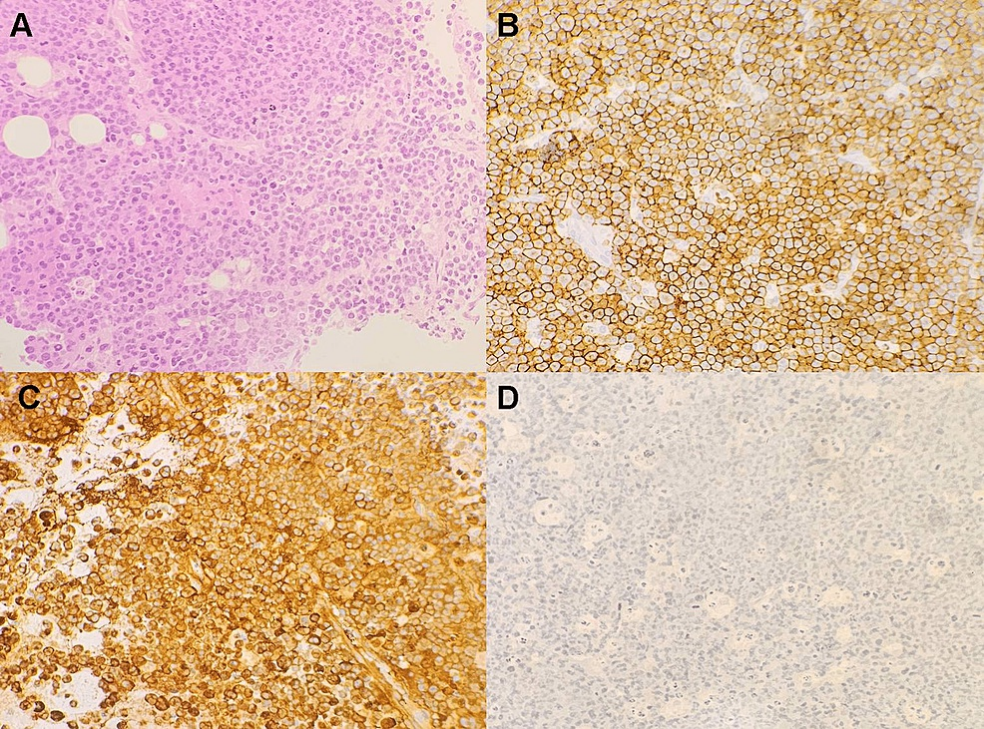
A: Tumor tissue composed of cleaved, multilobated, and monocytoid cells (hematoxylin-eosin stain, original magnification, X 400), B: diffuse membranous staining in tumor cells (CD38 stain, original magnification, X 400), C: diffuse staining in tumor cells (Lambda stain, original magnification, X 400), D: no staining of tumor cells (Kappa stain, original magnification, X 400).

Thinning and bone destruction due to the mass were observed in the clivus. Cerebral hemispheres were in normal structure. An endonasal endoscopic transsphenoidal approach was planned for the patient to obtain a sample from the mass. The removed material was sent to pathology with the preliminary diagnosis of chordoma. As a result of the histopathological description, a diagnosis of plasmacytoma was made (Figure 4). Since it would be difficult to remove the tumor after the diagnosis was confirmed, curative radiotherapy treatment was planned. A check-up was recommended after two weeks. During external cranial radiotherapy, a computed tomography was taken for the purpose of radiotherapy planning. A total of 4000 cGy treatment was applied in 20 fractions, with the fraction dose for the tumor area being 200 cGy. At the one-month follow-up after radiotherapy, the patient's vision and ptosis improved. The patient was investigated for multiple myeloma during this period. A bone marrow biopsy was performed. Maturation was normal in the bone marrow evaluation. Megakaryocytes were sufficient. No increase in plasma cells was observed. The absence of clonal plasma cell increase in the bone marrow and the presence of a single plasmacytoma were found to be compatible with clinical solitary plasmacytoma. Survival after radiotherapy was seven months. 

## Discussion

Plasmacytomas are extremely rare among the tumors seen in the clivus (<1%). It is often confused with the chordoma of the clivus. Chordomas, the most common tumor in this region, account for only 0.1-0.2% of all intracranial tumors [3]. We presented a case of clivus plasmacytoma that invaded the cavernous sinus and pituitary gland. It is reported in the literature that the most frequently affected cranial nerve in clivus tumors is the abducens nerve. Accordingly, diplopia is a common symptom [7]. In our case, plasmacytoma infiltrating the cavernous sinus caused ptosis by compressing the oculomotor nerve. Solitary plasmacytomas can mostly be seen in the vertebrae, calvaria, and appendicular skeleton. The most common location for skull base plasmacytoma is the nasopharynx. Sphenoid bone and clivus involvement is less common [8,9]. Anosmia, nasal congestion, recurrent nosebleeds, headaches, and vision-related problems can often be observed. It may also remain asymptomatic for a long time [10]. Optic nerve compression is a rare symptom secondary to a plasmocytoma and has been seldom reported in the literature [11,12]. In our case, there was also a significant increase in fluid in the dural sheath around the optic nerve, which is an indication of the pressure of the mass on the surrounding anatomical structures. Depending on the severity of the compression on the optic nerve, our patient also developed vision problems.

Intracranial tumors were staged by Sekhar [13] as I-V according to their location in the cavernous sinus and their relationship with the internal carotid artery. According to this classification, bilateral cavernous sinus involvement and the presence of a fully coiled internal carotid artery indicate stage 5. In our case, a plasmacytoma infiltrating the bilateral cavernous sinus and completely covering the internal carotid arteries was compatible with stage 5. Compared to other skull base masses, plasmacytomas also show higher signal intensity. Because plasmacytomas are often confused with chordomas, it is also important to know the radiological appearance of chordomas. The border between chordomas and clivus is usually non-sclerotic and sharp [14]. In differentiating between plasmocytomas and meningiomas, plasmocytomas show homogeneous contrast uptake similar to meningiomas in computed tomography examinations. They are often indistinguishable from meningiomas on MRI [1]. It has been reported that surgical treatment is the best option if the tumor can be completely removed. In cases where the entire mass cannot be removed, radiotherapy may be beneficial after surgery [15].

## Conclusions

The clival region has become one of the most challenging issues in neurosurgery with its complex anatomy and uniquely behaving tumors. Tumors that invade the cavernous sinus adjacent to this region are of great importance due to their proximity to the cranial nerves and internal carotid artery. Therefore, in order to perform a complete resection of tumors around the clivus, knowing the anatomy of this region will prevent possible complications. Plasmacytomas should not be kept in mind in the differential diagnosis of intracranial tumors. Localized radiotherapy for plasmacytoma is a good option, especially when the tumor cannot be removed by surgery.
